# Supplementary material for: Rhynchophorus palmarum (Linnaeus, 1758) (Coleoptera: Curculionidae): Guarani-Kaiowá indigenous knowledge and pharmacological activities
Source: PLoS One. 2021 Apr 29;16(4):e0249919. doi: 10.1371/journal.pone.0249919 (PMC8084164; doi:10.1371/journal.pone.0249919)
Supplement: S1 File — (PDF) [file pone.0249919.s001.pdf]

Rhynchophorus palmarum Linnaeus (Coleoptera: Curculionidae): GUARANI-KAIOWÁ INDIGENOUS KNOWLEDGE AND PHARMACOLOGICAL ACTIVITIES

Kellen Natalice Vilharva[1], Daniel Ferreira Leite[1], Helder Freitas dos Santos[1], Katia Ávila Antunes[1], Paola dos Santos da Rocha[1], Jaqueline Ferreira Campos[1], Claudiane Vilharroel Almeida[2], Maria Lígia Rodrigues Macedo[2], Denise Brentan Silva[3], Caio Fernando Ramalho de Oliveira[1,2], Edson Lucas dos Santos[1], Kely de Picoli Souza[1,*]

[1]Research Group on Biotechnology and Bioprospecting Applied to Metabolism, Federal University of Grande Dourados, Dourados, Brazil

[2]Laboratório de Purificação de Proteínas e suas Funções Biológicas, Federal University of Mato Grosso do Sul, Campo Grande, MS, Brazil

[3]Laboratory of Natural Products and Mass Spectrometry, Federal University of Mato Grosso do Sul, Campo Grande, MS, Brazil

[*]Corresponding author: tel: + 55 67 98132-8770, e-mail: kelypicoli@gmail.com

SUPPLEMENTARY MATERIAL

Supplementary material S1. Record of traditional Guarani-Kaiowá indigenous knowledge.

In the Guarani-Kaiowá community, the process of collecting the larvae, as well as preparing the oil, is carried out through a series of procedures, transmitted from generation to generation. *Rhynchophorus palmarum* larvae are collected by married women, most of the time. If a single woman from the village participates in the collection of the larvae, one of the older women involved in the collection performs a prayer to protect the single woman. According to the Guarani-Kaiowá tradition, a single woman who came to touch the larvae without praying could give birth to children with congenital problems, specifically with problems in bone formation. Besides, the collection of larvae and the preparation of oil must be done by the women of the village and, most of the time, in silence. These women, usually the mothers of the families, are responsible for preparing various medicines in the village. In the case of the obtainment of oil from *R. palmarum* larvae, the process is made on the fire, where the larvae are stirred until the oil releasing. At the end of the heating process, the larvae oil and carcasses are placed in a glass container and stored in a dark place.

Knowledge and practices in an indigenous community are transmitted and give life to the indigenous way of life. Some of the Guarani-Kaiowá secular knowledge is passed on to the youngest by *Maxuypy*, the matriarch. Among the knowledge transmitted by *Maxuypy*, is the use of natural products for therapeutic purposes. The application of animal fat in the treatment of diseases is known, such as anaconda fat, used to treat back pain; wolf fat used to treat bronchitis; armadillo fat used to treat asthma and sinusitis; and several other animals that have not yet been registered. In the community of ‘Terra Indígena Takuara’, located in the municipality of Juti, Mato Grosso do Sul, one of the many knowledge about the use of animals mentions the use of *Mbuku kyra*. *Mbuku* is an entity left by the divinity in the Earth. The legend about his creation is part of the sacred story of the creation of indigenous life, which tells the origin of some sacred animals. The following story is a small part of the story *Ara rehegua* (literal translation “legend about time”), made by *Maxuypy* Julia Cavalheira (76 years old), carried out around the fire in the Takuara Indigenous Land. This is a long story, told over days, that describes the origin of several animals. The following report deals with the creation of Mbuku, the insect used by the Guarani-Kaiowá:

“One day, the brothers *Paikwara* (Sun) and *Jasyete* (Moon) were walking near an abyss. They were starving. Then Paikwara spotted a banana tree and said to Jasyete:

- Wait for me here, I will get some bananas for us. *Paikwara* took the bananas very carefully to avoid awakening the *Pytumbory*, an evil entity, equivalent to a demon, which rested next to the banana tree. After consuming the bananas collected by *Paikwara*, *Jasyete* said:

- I want to get more bananas!

"Do not go, *Jasyete*!" Said Paikwara. *Pytumbory* is ready to drop you into the abyss”. *Jasyete* did not listen to his sister's recommendation and went to collect more bananas. As *Jasyete* prepared to collect the biggest bananas, *Pytumbory* knocked him over the edge. When witnessing the scene, *Paikwara* cried a lot for *Jasyete's* death and stood there, desolate, looking into the abyss, while *Pytumbory* left. Shortly thereafter, *Mberuypy*, the queen deity of the *Mberu*, who would later give rise to flies. *Mberuypy* then asked *Paikwara*:

- Why is our older brother sad? *Paikwara* replied:

- My younger brother (*Jasyete*) fell into the abyss, it was *Pytumbory* who knocked him down! Then, the *Mberu* claimed that they would bring their brother back. Paikwara warned *Mberuypy* that the *Mberu* should collect his brother's *Kurusuyta*, a fragment of *Jasyete's* collarbone bone, as *Paikwara* knew that *Jasyete's* death had been a long time. With his brother's *Kurusuyta*, *Paikwara* could resurrect him. The *Mberu* descended the abyss and, soon after, returned with *Jasyete's* entrails. Because *Mberu* did not return with *Jasyete's Kurusuyta*, *Paikwara* was very sad and ordered that, from that moment on, all *Mberu* would live on remains, and blew on them. Thus, blowflies arrived.

Other animals tried to seek *Jasyete's Kurusuyta* until one-day *Kykyin* (mother or queen of the larvae of the coconut tree) approached *Paikwara* and asked her why she was so sad. *Paikwara* reported what happened to his brother *Jasyete*. *Kykyin* said he could find *Jasyete's Kurusuyta*. So, *Kykyin* descended the abyss and, after a long time, returned with the remains of *Jasyete's* brain. *Paikwara* once again

cried and said he needed *Jasyete's Kurusuyta*. Then, *Kykyin* descended the chasm again in search of *Jasyete's Kurusuyta*, to help *Paikwara* get his brother back. After a long time, Kykyin returned, without success, again with the remains of *Jasyete's* brain. At that moment, *Paikwara* acknowledged *Kykyin's* effort in trying to help him resurrect his brother. So, he sent a blessed breath on him, saying the following words:

- That their children survive only within the *Pindó* and *Mbokaja* (coconut species). That your children serve as food for all my descendants and serve as a cure for those who wander the earth! Thus, as a way of thanking *Kykyin's* efforts, *Mbuku* came into being to life".

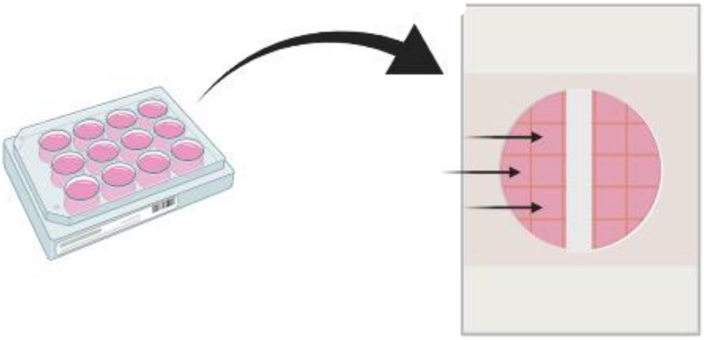

Supplementary Figure S2. Scheme for the acquisition of images of the microplate wells. The well, shown in detail in the figure (on the right), shows the position where the images were taken (black arrows). The clear vertical line shown in the well represents the removal of the adhered cells, carried out with the aid of a 1000 µl tip. Image prepared on the BioRender.com website.
