# Supplementary material for: Rhynchophorus palmarum (Linnaeus, 1758) (Coleoptera: Curculionidae): Guarani-Kaiowá indigenous knowledge and pharmacological activities
Source: PLoS One. 2021 Apr 29;16(4):e0249919. doi: 10.1371/journal.pone.0249919 (PMC8084164; doi:10.1371/journal.pone.0249919)
Supplement: S2 File — (DOCX) [file pone.0249919.s002.docx]

**Financial Disclosure section**

*The funders had no role in study design, data collection and analysis, decision to publish, or preparation of the manuscript. The authors received no specific funding for this work.*
