# Supplementary material for: Rhynchophorus palmarum (Linnaeus, 1758) (Coleoptera: Curculionidae): Guarani-Kaiowá indigenous knowledge and pharmacological activities
Source: PLoS One. 2021 Apr 29;16(4):e0249919. doi: 10.1371/journal.pone.0249919 (PMC8084164; doi:10.1371/journal.pone.0249919)
Supplement: Attachment — Submitted filename: Revision_PONE-D-20-36667.doc [file pone.0249919.s003.doc]

The manuscript (PONE-D-20-36667) entitled "*Rhynchophorus palmarum* Linnaeus (Coleoptera: Curculionidae): Guarani-Kaiowá indigenous knowledge and pharmacological activities" is about the therapeutic use of oils obtained from *R. palmarum* larvae. The authors determined the chemical characteristics of the obtained oil, and, for the first time, ~~are~~ described its antioxidant and healing properties. They paid a special attention to the Guarani-Kaiowá ethnic community and their knowledge of zootherapy. Submitted manuscript contains some valuable information, so, this interesting research deserves attention having in mind a possibility of vanishing of traditional knowledge and practices over time. Also, the presented results may be of some help in the future development of various pharmaceutical products.

Unfortunately, I cannot recommend accepting the manuscript in its present form.

Following are some comments and questions raised during the review of the text.

Significant revision of English scientific writing is needed.

**ABSTRACT**

Abstract is concise and contains factual information.

**INTRODUCTION**

In the Introduction, the authors stated the objectives of their work that are supported by appropriate facts.

**MATERIALS AND METHODS**

The experiments are well designed and performed. But, some parts of Material and Methods section need a bit more details. Specifically, these issues are highlighted:

***Obtaining R. palmarum larvae oil***

In Supplementary material S1, the method of collecting larvae is already described in detail.

Line 93-94	The authors mentioned that the larvae were collected in Takuara village.

*When this procedure was conducted? How many larvae were collected?*

Line 98	“...larvae were washed with distilled water, dried with paper towels, weighed, and frozen...“

*What were the temperatures that larvae were kept on, until the beginning of the experimental procedure?*

***Determination of the chemical composition of RPLO***

Line 124	“...followed by stirring and incubation of the mixture“

*The time of incubation?*

***Antioxidant activity of RPLO***

*In formula for DPPH percentage, Abs sample is the absorbance of the sample and Abs control is the absorbance of control? Please, provide clarifications.*

Line 149-150	“The results of three experiments performed in triplicate were used to determine the antioxidant activity.“

*How many samples were in each experiment?*

***Antimicrobial activity of RPLO***

Line 176	“The experiments were carried out in triplicate. “

*How many samples were in each of this triplicates?*

***Cell culture, viability, and migration***

*Please, provide clarifications for Abs treated cells and Abs control cells.*

***Statistical analysis***

*Parametric statistics should be used when the assumptions of the models (ANOVA) can be reasonably met. A test of homogeneity of variances (for example, Levene test for homogeneity of variances) must show that in all cases variances were not significantly heterogeneous, as well as a Kolmogorov-Smirnov test for normal distribution fitting. So, the authors should test data distribution by, for example, Kolmogorov-Smirnov test and point it out in Statistical analysis section. This is very important because normal distribution is prerequisite for using ANOVA test.*

**RESULTS**

***Antimicrobial activity of the RPLO***

*Please, see the Table 3. Did you mean MIC and MBC, instead of CIM and MBC?*

***Effect of RPLO on the viability of MRC-5 cells***

*Maybe, it would be better to rephrase this section. E.g., at given concentrations, cell viability was in what range? Compare to the control, which concentration of RPLO the most changes the viability of MRC-5 cells?*

*In legend of Fig 4, please mention the number of samples. n= 9?*

***Effect of RPLO on the migration rate of MRC-5 cells***

*Line 299*	*"As shown in Fig 5A…" instead of "As shown in Fig 5…"*

*Line 302-304	Please, rephrase the sentence. Maybe, something like the following:* *The cell migration rate was statistically increased by 60% in the wells treated with RPLO compared to the wells receiving the control treatment (Fig. 5B).*

*Figure 5. Legend should be rewritten. It should be as clear and concise as possible. In addition, please mention the number of samples. Was it 9?*

***Toxicity of RPLO in a C. elegans model***

*In legend of Fig 6, please mention the number of samples. n= 10?*

**DISCUSSION**

Please improve this section. The discussion should be clearly and concisely presented. Namely, the same things are repeated in several paragraphs – e.g. see lines 369-371 and 388-391; lines 402-408 and 418-425…

**REFERENCES**

Please, check this section once again.
