# Supplementary material for: Rhynchophorus palmarum (Linnaeus, 1758) (Coleoptera: Curculionidae): Guarani-Kaiowá indigenous knowledge and pharmacological activities
Source: PLoS One. 2021 Apr 29;16(4):e0249919. doi: 10.1371/journal.pone.0249919 (PMC8084164; doi:10.1371/journal.pone.0249919)
Supplement: Attachment — Submitted filename: Response to reviewers.doc [file pone.0249919.s004.doc]

Dear editor,

We are very grateful for the comments raised during the review process. We would like to make it clear that all suggestions have been analyzed and the manuscript has been modified. The responses to each survey were entered as "> response". In the responses, the indicated lines correspond to the file "Revised Article with Changes Highlighted".

The manuscript (PONE-D-20-36667) entitled "*Rhynchophorus palmarum* Linnaeus (Coleoptera: Curculionidae): Guarani-Kaiowá indigenous knowledge and pharmacological activities" is about the therapeutic use of oils obtained from *R. palmarum* larvae. The authors determined the chemical characteristics of the obtained oil, and, for the first time, ~~are~~ described its antioxidant and healing properties. They paid a special attention to the Guarani-Kaiowá ethnic community and their knowledge of zootherapy. Submitted manuscript contains some valuable information, so, this interesting research deserves attention having in mind a possibility of vanishing of traditional knowledge and practices over time. Also, the presented results may be of some help in the future development of various pharmaceutical products.

Unfortunately, I cannot recommend accepting the manuscript in its present form.

Following are some comments and questions raised during the review of the text.

Significant revision of English scientific writing is needed.

**ABSTRACT**

Abstract is concise and contains factual information.

**INTRODUCTION**

In the Introduction, the authors stated the objectives of their work that are supported by appropriate facts.

**MATERIALS AND METHODS**

The experiments are well designed and performed. But, some parts of Material and Methods section need a bit more details. Specifically, these issues are highlighted:

***Obtaining R. palmarum larvae oil***

In Supplementary material S1, the method of collecting larvae is already described in detail.

Line 93-94	The authors mentioned that the larvae were collected in Takuara village.

*When this procedure was conducted? How many larvae were collected?*

Line 98	“...larvae were washed with distilled water, dried with paper towels, weighed, and frozen...“

*What were the temperatures that larvae were kept on, until the beginning of the experimental procedure?*

*> In this version of the manuscript we added further information regard the process of oil obtainment, informing the number of larvae, temperature of storage and further details requested (line 98-99).*

***Determination of the chemical composition of RPLO***

Line 124	“...followed by stirring and incubation of the mixture“

*The time of incubation?*

*> We inserted in this version of the manuscript the time of incubation, of 15 min (line 125).*

***Antioxidant activity of RPLO***

*In formula for DPPH percentage, Abs sample is the absorbance of the sample and Abs control is the absorbance of control? Please, provide clarifications.*

*> We appreciate this observation and now, we included more information about the formula (line 148).*

Line 149-150	“The results of three experiments performed in triplicate were used to determine the antioxidant activity.“

*How many samples were in each experiment?*

*> This was another important observation raised by referee. We included more details about this topic, informing that three independent assays. In each assay, we used triplicates for each oil concentration (n=3). Thus, each assay was carried out with n= 3. The sum of three independent assays resulted in a total samples of n= 9 (line 152-153).*

***Antimicrobial activity of RPLO***

Line 176	“The experiments were carried out in triplicate. “

*How many samples were in each of this triplicates?*

*> In each assay, we used triplicates for each oil concentration (n=3). Thus, each assay was carried out with n= 3. The sum of three independent assays resulted in a total samples of n= 9 (3 assays using a 3 samples) (line 180).*

***Cell culture, viability, and migration***

*Please, provide clarifications for Abs treated cells and Abs control cells.*

*> We appreciate this observation and now, we included more information about the formula (line 206).*

***Statistical analysis***

*Parametric statistics should be used when the assumptions of the models (ANOVA) can be reasonably met. A test of homogeneity of variances (for example, Levene test for homogeneity of variances) must show that in all cases variances were not significantly heterogeneous, as well as a Kolmogorov-Smirnov test for normal distribution fitting. So, the authors should test data distribution by, for example, Kolmogorov-Smirnov test and point it out in Statistical analysis section. This is very important because normal distribution is prerequisite for using ANOVA test.*

*> We thank the referee regards this so important observation. In this version of the manuscript, we carried out further statistics analyses, such as suggested. So, we included the test of homogeneity of variances (Levene) and for normal distribution (KS test). Since non-significant values were obtained, we continued using ANOVA (line 242-244).*

**RESULTS**

***Antimicrobial activity of the RPLO***

*Please, see the Table 3. Did you mean MIC and MBC, instead of CIM and MBC?*

*> These mistakes were corrected.*

***Effect of RPLO on the viability of MRC-5 cells***

*Maybe, it would be better to rephrase this section. E.g., at given concentrations, cell viability was in what range? Compare to the control, which concentration of RPLO the most changes the viability of MRC-5 cells?*

*> This section was rewritten to improve the clarity (line 294-298).*

*In legend of Fig 4, please mention the number of samples. n= 9?*

*> The mention regards the n=9 was included (line 302).*

***Effect of RPLO on the migration rate of MRC-5 cells***

*Line 299*	*"As shown in Fig 5A…" instead of "As shown in Fig 5…"*

*Line 302-304	Please, rephrase the sentence. Maybe, something like the following:* *The cell migration rate was statistically increased by 60% in the wells treated with RPLO compared to the wells receiving the control treatment (Fig. 5B).*

*> Based in observation of referee, we modified the section, improving the clarity of suggested sentences (line 306-310).*

*Figure 5. Legend should be rewritten. It should be as clear and concise as possible. In addition, please mention the number of samples. Was it 9?*

*> Actually, the legend contained excessive sentences. This mistake was corrected and the number of samples was included in this version (line 314-322).*

***Toxicity of RPLO in a C. elegans model***

*In legend of Fig 6, please mention the number of samples.* ***n= 10?***

*> This was a good observation. This assay contains a n= 90, formed by triplicates containing 10 worms/well (n= 30). Once the assay was carried out three times independently, the number of 90 was obtained (line 332).*

**DISCUSSION**

Please improve this section. The discussion should be clearly and concisely presented. Namely, the same things are repeated in several paragraphs – e.g. see lines 369-371 and 388-391; lines 402-408 and 418-425…

*> We included modifications in discussion topic in order to make more conscious. However, in some points different approaches were necessary. So, whenever possible we improved the section.*

**REFERENCES**

Please, check this section once again.

*> We made corrections in references section.*
