# Supplementary material for: Rhynchophorus palmarum (Linnaeus, 1758) (Coleoptera: Curculionidae): Guarani-Kaiowá indigenous knowledge and pharmacological activities
Source: PLoS One. 2021 Apr 29;16(4):e0249919. doi: 10.1371/journal.pone.0249919 (PMC8084164; doi:10.1371/journal.pone.0249919)
Supplement: Attachment — Submitted filename: PONE-D-20-36667R2_R2.doc [file pone.0249919.s005.doc]

The manuscript PONE-D-20-36667R2 entitled “*Rhynchophorus palmarum* (Linnaeus, 1758) (Coleoptera: Curculionidae): Guarani Kaiowá indigenous knowledge and pharmacological activities” was reviewed and the authors have addressed all the issues that reviewers had raised. I suggest that the manuscript should be accepted after an additional minor revision.

Minor comment:

The Material and Methods section has been greatly improved. But, Statistical analysis description could be a bit more precise, something like this:

The data were expressed as mean ± standard error of the mean (SEM). Prior to the statistical analysis, Levene´s test of homogeneity of variances was used to assume that variances are equal across groups or samples and Kolmogorov-Smirnov test was used to assess the normality of the data. The mean values of the cell migration rate were analyzed by One-way analysis of variance (ANOVA) followed by Dunnett's post-test. In *in vivo* assays, a t-test was used to determine differences between groups. All analyses were performed using GraphPad Prism 5 software. The results were considered to be statistically significant at p ≤ 0.05.

Also, Figures legends would be rewritten, e.g.

Fig 3. Viability of MRC-5 cells incubated with different concentrations of RPLO for 24 h. The graph shows the means ± SEM of three independent experiments performed in triplicate (n= 9). The observed changes were not statistically significant (t-test).

Fig 4. The effect of RPLO on the migration rate of MRC-5 cells. (A) Representative image from assay. The wound areas were determined at 0 h and after 24 h of incubation. Control cells were incubated with culture media. Cells incubated with 0.5% of oil were named RPLO. Scale bar is 1 mm. (B) The graph shows the means ± SEM of three independent experiments performed in triplicate (n= 9). An asterisk (*) indicate statistically significant differences between mean values (one-way ANOVA, Dunnett's post-test, p <0.05).

Fig 5. Acute toxicity of RPLO in the *C. elegans* experimental model *in vivo.* Viability was recorded at (A) 24 h and (B) 48 h of treatment. The graph shows the means ± SEM of three independent experiments performed in triplicate (n= 90). The observed changes were not statistically significant (t-test).
