# Supplementary material for: Rhynchophorus palmarum (Linnaeus, 1758) (Coleoptera: Curculionidae): Guarani-Kaiowá indigenous knowledge and pharmacological activities
Source: PLoS One. 2021 Apr 29;16(4):e0249919. doi: 10.1371/journal.pone.0249919 (PMC8084164; doi:10.1371/journal.pone.0249919)
Supplement: Attachment — Submitted filename: Response to Reviewers.doc [file pone.0249919.s006.doc]

The manuscript PONE-D-20-36667R2 entitled “*Rhynchophorus palmarum* (Linnaeus, 1758) (Coleoptera: Curculionidae): Guarani Kaiowá indigenous knowledge and pharmacological activities” was reviewed and the authors have addressed all the issues that reviewers had raised. I suggest that the manuscript should be accepted after an additional minor revision.

*We would like to thank Dr. José Antonio González for taking care of the smallest details that will certainly enrich our manuscript. Below are the responses to the surveys, which are marked in blue.*

Minor comment:

The Material and Methods section has been greatly improved. But, Statistical analysis description could be a bit more precise, something like this:

The data were expressed as mean ± standard error of the mean (SEM). Prior to the statistical analysis, Levene´s test of homogeneity of variances was used to assume that variances are equal across groups or samples and Kolmogorov-Smirnov test was used to assess the normality of the data. The mean values of the cell migration rate were analyzed by One-way analysis of variance (ANOVA) followed by Dunnett's post-test. In *in vivo* assays, a t-test was used to determine differences between groups. All analyses were performed using GraphPad Prism 5 software. The results were considered to be statistically significant at p ≤ 0.05.

*> We appreciate the suggestion, in this version the manuscript the section Statistical analysis was rewritten observing the improvement of clarity.*

Also, Figure legends would be rewritten, e.g.

Fig 3. Viability of MRC-5 cells incubated with different concentrations of RPLO for 24 h. The graph shows the means ± SEM of three independent experiments performed in triplicate (n= 9). The observed changes were not statistically significant (t-test).

Fig 4. The effect of RPLO on the migration rate of MRC-5 cells. (A) Representative image from assay. The wound areas were determined at 0 h and after 24 h of incubation. Control cells were incubated with culture media. Cells incubated with 0.5% of oil were named RPLO. Scale bar is 1 mm. (B) The graph shows the means ± SEM of three independent experiments performed in triplicate (n= 9). An asterisk (*) indicate statistically significant differences between mean values (one-way ANOVA, Dunnett's post-test, p <0.05).

Fig 5. Acute toxicity of RPLO in the *C. elegans* experimental model *in vivo.* Viability was recorded at (A) 24 h and (B) 48 h of treatment. The graph shows the means ± SEM of three independent experiments performed in triplicate (n= 90). The observed changes were not statistically significant (t-test).

*> The same care regards the previous appointment was took in consideration, in order to become the Figure legends clear.*
